# Ferric-loaded lipid nanoparticles inducing ferroptosis-like cell death for antibacterial wound healing

**DOI:** 10.1080/10717544.2022.2152134

**Published:** 2022-12-01

**Authors:** Ying Zhou, Chong-Yang Cai, Cheng Wang, Guo-Ming Hu, Yu-Ting Li, Meng-Jiao Han, Shen Hu, Pu Cheng

**Affiliations:** a Department of Gynecology, Second Affiliated Hospital, Zhejiang University School of Medicine, Hangzhou, China; b Department of Urology, Hangzhou TCM Hospital Affiliated to Zhejiang Chinese Medical University, Hangzhou, China; c School of Pharmacy, Changzhou University, Changzhou, China; d Department of General Surgery (Breast and Thyroid Surgery), Shaoxing People's Hospital (Shaoxing Hospital, Zhejiang University School of Medicine), Zhejiang, China; e Department of Nephrology, Sir Run Run Shaw Hospital, Zhejiang University School of Medicine, Hangzhou, China; f Department of Obstetrics, Second Affiliated Hospital, Zhejiang University School of Medicine, Hangzhou, China; g Key Laboratory of Tumor Microenvironment and Immune Therapy of Zhejiang Province

**Keywords:** Skin infection, lipid nanoparticles, ferroptosis, antibacterial, wound healing

## Abstract

Skin infection is a major health issue that usually is caused by the continuous proliferation of bacteria in wounds. With the abuse of antibiotics worldwide, the battle against skin infection is becoming more and more difficult. Therefore, the development of new ways with different antibacterial mechanisms to current antibiotics is urgently needed. Inspired by the powerful inhibition of ferroptosis used in cancer therapy, here in our study, ferric-loaded lipid nanoparticles (Fe-LNPs) with unform size (∼130 nm) and surface charge (∼12 mV) were constructed and found to effectively inhibit the growth of both Gram positive (Staphylococcus aureus, *S. aureus*) and negative (Escherichia coli, *E. coli*) strains, possibly due to induction of ferroptosis-like cell death mechanisms. Most importantly, Fe-LNPs can also effectively inhibit the proliferation of *S. aureus* in a skin infection model and promote the healing of wounds. The Fe-LNPs can be applied as a powerful antibacterial formulation for future application in clinic.

## 1. Introduction

Skin infection, characterized by the invasion of microbe to the skin layers and the related soft tissues, is commonly happens in all healthcare settings (Esposito et al., 2016). It was reported that there were over 2.3 million cases of skin infection between 2005 and 2010 in USA alone (Miller et al., 2015). Therefore, the burden and cost raised by skin infection, including ambulatory visits and hospitalizations, are substantial all over the world (Kaye et al., 2019). In recent decades, with the wide abuse of antibiotics across the globe, the emerging of super bacteria that can survive under the challenge of one or more antibiotics, is posing potential threat to human health and brings additional difficulties to the well management of skin infection (Romero et al., 2012; Bassetti et al., 2022). Therefore, there is an urgent need to find other ways to solve the acquired drug resistance of super bacteria to prevent potential pandemics.

In recent years, many new ways that can effectively inhibit the growth of bacteria including antimicrobial peptide-based therapy (C. Wang et al., 2021), photo-based therapy (photodynamic/photothermal) (Huo et al., 2021; Huang et al., 2022; Songca & Adjei, 2022), and catalytic therapy (Y. Li et al., 2021) are explored. Among which the use of reactive oxygen species (ROS) to kill bacteria is generally recognized as a promising way, as it mimics the way mammalian cells to fight against bacterial infections (Nathan et al., 1979) and usually induce low potential of resistance on bacteria (Ren et al., 2020). The use of ROS-based strategies for antibacterial applications have been widely practiced by many previous studies and have shown preferable inhibition on various strains (Song et al., 2019; Qiu et al., 2021), even super bacteria (Hamida et al., 2020).

Ferroptosis is a recently discovered type of cell death. It was usually associated with accumulation of iron and lipidic ROS, which results in a decrease in antioxidant capacity and finally leading to oxidative cell death (J. Li et al., 2020; D. Tang & Kroemer, 2020). During this process, the fenton reaction that characterized as free radical reactions catalyzed by ferrous ions plays an important role for the trigger of ferroptosis (B. Wang et al., 2020). Therefore, the delivery of iron is widely recognized as a suitable way to initiate the ferroptosis of target cells (L. Chen et al., 2019; Bilal et al., 2021). Accordingly, the use of ferroptosis to combat cancers are widely reported with good therapeutic outcomes (Z. Shen et al., 2018; Xiong et al., 2021). It was reported by previous study that bacteria usually have lower level of intracellular reducing substance (X. Shen et al., 2020), which makes them more susceptible to ROS-based therapies, including ferroptosis. Jiang and coworkers have employed ferric ammonium citrate (FAC) loaded liposome as iron-dependent lipid peroxide generator to combat cancer (He et al., 2020). However, the application of ferroptosis-related therapies on antibacterial fields is rarely reported which deserves more explorations.

Lipid nanoparticles (LNPs) represent a kind of highly biocompatible and accessible carrier for drug delivery (Yuan et al., 2008; Zhao et al., 2018; Dolatabadi et al., 2021). In the past decades, the application of LNPs have extended to many fields including medical therapy (Rahat et al., 2021; Yang et al., 2021), plant science (Fincheira et al., 2020), food chemistry (da Silva Santos et al., 2019), and other innovative areas such as nanoreactors (Tewari et al., 2022). In particular, LNPs have been successfully adopted in drug delivery for the combat of different disease (Tenchov et al., 2021). More importantly, LNPs also serves as the star candidate for the recent development of mRNA-based vaccines to combat COVID-19 (Hou et al., 2021; Wilson & Geetha, 2022). Compared with other parallel carriers, LNPs can satisfy the large-scale production in facile ways, which have went through the challenges for marketing (Kraft et al., 2014). Therefore, using LNPs as drug delivery carrier for the delivery of iron to trigger ferroptosis like cell death for antibacterial application might be a promising strategy.

Inspired by the aforementioned researches, here in this study, we used FAC as the iron source and load it with LNPs. The antibacterial performance finally prepared nanoparticle (short for Fe-LNPs) was tested on both Gram positive (Staphylococcus aureus, *S. aureus*) and negative (Escherichia coli, *E. coli*) strains in vitro. Finally, a *S. aureus* infected wound model was established to evaluate the in vivo antibacterial profile of Fe-LNPs.

## 2. Material and methods

### 2.1. Materials and apparatus

FAC was purchased from Yuanlong food ingredients Co., Ltd. (Cangzhou, China). Phosphatidylcholine (PC) was purchased from Aladdin (Shanghai, China). Absolute ethanol was purchased from Titan Scientific Co., Ltd. (Shanghai, China). Lecithin was purchased from Aladdin Co., Ltd. (Shanghai, China). The Live/Dead bacterial vitality kit was purchased from Solarbio (Beijing, China). *S. aureus* (ATCC 6538) and *E. coli* (ATCC 8739) were purchased from ATCC. Peptone soybean broth (TSB), trypticase soy agar medium (TSA), lysogeny broth (LB), and LB agar were purchased from Hangzhou Microbial Reagent Co., Ltd (Hangzhou, China). All chemicals with specific statement were of analytical grade from Titan Scientific Co., Ltd. (Shanghai, China) and used without other treatments. Deionized water (Millipore, USA) was used in all experiments.

Magnetic stirrer (SY18-type 1, Sile Instrument Co., Ltd., Shanghai, China) was used in all experiments requiring agitation. The size and zeta potential were determined by Zetasizer Nano (Malvern, UK). The sonication was performed using prob type ultrasonic instrument and centrifugation was performed using TGL-16M (Cence, Changsha, China). The UV absorbance and spectrum were recorded by UV spectrophotometer (UV-3600, Shimadzu, Japan). The bacterial concentration was recorded using a microplate reader (Synergy NEO, BioTek, Vermont, USA). A thermostatic shaker (THZ-300C, Yiheng Scientific Instruments Co., Ltd, Shanghai, China), Superclean bench (SW-CJ-2FD, Sujing Antai Co., Ltd, Suzhou, China), and biochemical incubator (SPX-150BSH-type II, CIMO Medical Device Manufacturing Co., Ltd, Shanghai, China) were used to culture bacteria. The fluorescence imaging were imaged by inverted fluorescence microscope (Eclipse Ti-S, Nikon, Japan). Deionized water was obtained from water purification machine (H20pro-UV-T, Millipore, USA).

### 2.2. Preparation of Fe-LNPs

The ferric-loaded lipid nanoparticles (Fe-LNPs) were prepared with reference to previously reported protocol with some modifications (He et al., 2020). In brief, FAC (7.5 mg) was dissolved in glass containing 2 mL of deionized water while 3 mg of PC was dissolved in another glass containing 1 mL of ethanol. The lipid solution was injected into the FAC solution under stirring and the reaction was allowed to proceed under room temperature for 30 min. After being sonicated for 30 min (50%, 250 W, 3 s/3 s) in ice bath, the solution was subjected to dialysis (WUCO = 14,000) to remove ethanol and unloaded FAC. Free LNPs were prepared using the same protocol without the addition of FAC and subjected to dialysis to remove the remaining ethanol. The finally obtained nanoparticle solution was collected and store at 4 °C until further use.

### 2.3. Bacteria culture

Single bacterial colony of *S. aureus* and *E. coli* was transferred from agar plate to 10 mL of TSB and LB medium, respectively, and maintained in the thermostatic shaker for 12 h (250 rpm, 37 °C). The obtained bacteria solution was diluted and again cultured under the same condition for two more generations to give optimal viability of cells and then employed in the following studies (C. Wang et al., 2022).

### 2.4. *In vitro* antibacterial experiment

The *S. aureus* and *E. coli* solution at the OD_600_ value of 1.2 and 0.8, respectively was diluted by corresponding culture medium to give OD_600_ of 0.1. Then, 200 μL of the diluted bacteria solution was mixed with 200 μL of PBS (pH = 7.4, 10 mM) with or without different concentrations of LNPs, FAC, and Fe-LNPs (final concentrations of 1, 2, 3, 4 and 5 mM) and maintained in the thermostatic shaker for 1 h (250 rpm, 37 °C). The group diluted with PBS was named as 'PBS' while that diluted with PBS containing LNPs, FAC, and Fe-LNPs was named as 'LNPs, 'FAC', and 'Fe-LNPs', respectively. Afterward, the mixture was diluted with corresponding culture medium for 2 × 10^4^ times and then 100 μL of the diluted medium was transferred to TSA and LB agar plates, respectively. The plates were maintained in biochemical incubator (250 rpm, 37 °C) for another 18 h and then subjected to colony counting assay (Z. Zhang et al., 2022).

### 2.5. Live/dead staining

The *S. aureus* and *E. coli* solution at the density of 10^9^ CFU/mL was withdrawn (300 μL) and mixed with 100 μL of PBS with or without LNPs, FAC, and Fe-LNPs (final concentration of 4 mM) maintained in the thermostatic shaker for 1 h (250 rpm, 37 °C).Afterwards the mixture was centrifuged (5000 rpm, 10 min) and the obtained cells were subjected to live/dead staining using Syto9/PI according to the manufacturer's instructions, followed by imaging using inverted fluorescence microscope.

### 2.6. Biofilm formation inhibition and destruction studies

For biofilm inhibition study, 96 well-plate was loaded with 200 μL of PBS with or without LNPs, FAC, and Fe-LNPs (final concentration of 4 mM) and added with 200 μL of *S. aureus* and *E. coli* solution (10^9^ CFU/mL). The plates were placed in the incubator for 48 h and then added with 100 μL of crystal violet for biofilm staining. The crystal violet in the biofilm was then dissolved with 200 μL 80% ethanol in thermostatic shaker for 2 h (250 rpm, 37 °C) and the absorbance at 590 nm was determined using microplate reader.

For biofilm destruction study, 96-well plate was first loaded with 200 μL of corresponding culture medium with 2 μL of *S. aureus* and *E. coli* solution (10^9^ CFU/mL). The plates were placed in the incubator for 48 h and then replaced with 200 μL of PBS with or without LNPs, FAC, and Fe-LNPs (final concentration of 4 mM) and returned to the incubator for another 1 h. The medium was then replaced with 100 μL of crystal violet for biofilm staining. The crystal violet in the biofilm was then dissolved with 200 μL of 80% ethanol in thermostatic shaker for 2 h (250 rpm, 37 °C) and the absorbance at 590 nm was determined using microplate reader (Y. Li et al., 2022).

### 2.7. Bacterial growth curve

To study the inhibition details of Fe-LNPs on the growth of bacteria, the growth curve was plotted by monitoring the OD_600_ of solution at pre-determined time points. In brief, 96-well plate was loaded with 200 μL of bacteria solution at the concentration of 10^5^ CFU/mL and then added with 10 μL of PBS with or without LNPs, FAC, and Fe-LNPs (final concentration of 4 mM). The plates were placed in thermostatic shaker for 12 h (250 rpm, 37 °C) and the OD_600_ of each well was monitored every 1 h using microplate reader.

### 2.8. Effect of vitamin C and lipoic acid on antibacterial effect of Fe-LNPs

The 96-well plate was loaded with 200 μL *S. aureus* bacteria solution (OD_600_ of 0.1) containing different concentrations of vitamin C (VC) and lipoic acid (LA) (final concentrations of 0, 100, 200, 300, and 400 μM) with fixed Fe-LNPs concentration of 4 mM. The plates were placed in thermostatic shaker for 16 h (250 rpm, 37 °C) and the OD_600_ of each well was monitored using microplate reader.

### 2.9. Establishment and treatment of a mouse wound model *in vivo*

Female Balb/c mice (∼6 weeks and 20 g) were purchased from Cavens animal center (Changzhou, China) and were kept in SPF house of the center with free access to diet. All animal experiments were conducted under the supervision of Animal Ethics Committee of Changzhou and strictly followed the NIH guidelines for care and use of experiment animals.

To establish wound infection model, the skin on the back of anesthesized mice were damaged using a puncher to produce elliptic wounds (long axis of 10 mm and short axis of 6 mm). The wounds were seeded with 50 μL of *S. aureus* (10^8^ CFU/mL) for three times and then waited for 2 days for successful wound infection.

Mice with wound infection were randomly divided into three groups (*n* = 5) and subjected to treatment with PBS, 2 mM of Fe-LNPs (IC50), and 4 mM of Fe-LNPs (IC90) for every day for three times. The start of experiment was defined as Day 1 and the changes in wounds and body weights were recorded every day for 9 days. In addition, to assess the bacteria density at the wounds, the wound form each group was homogenated in sterile PBS and the bacteria were extracted in thermostatic shaker for 4 h (250 rpm, 37 °C). The extracted solution (100 μL) was transferred to TSA agar plate for another18 h of incubation in the incubator, followed by colony counting and imaging.

## 3. Results and discussion

**Figure 1.**
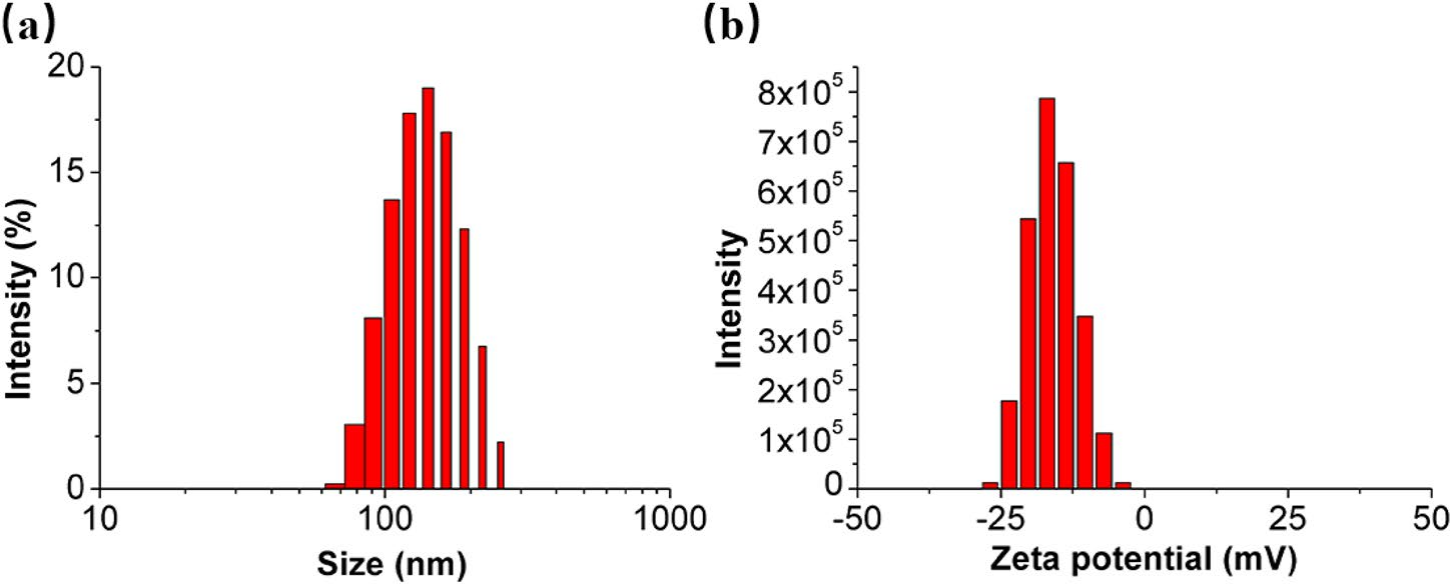
The particle size distribution (a) and zeta potential distribution (b) of the prepared Fe-LNPs.

Fe-LNPs were prepared using solvent-diffusion method, during which the LNPs can self-assemble into nanosized particles and the ferric ion was either encapsulated within or adsorbed to the LNPs. The prepared Fe-LNPs were characterized by evaluating their particle size and zeta potential distributions. The results are presented in Figure 1. It is shown that Fe-LNPs are nanosized formulations with good dispersion with majority size distribution at around 130 nm (Figure 1(a)). The zeta potential results in Figure 1(b) suggest that Fe-LNPs have negatively charged surface with average value of –12 mV. This is in line with previous reported LNPs formed by PC, which also suggests the successful preparation of Fe-LNPs (H. Zhang et al., 2022).

**Figure 2.**
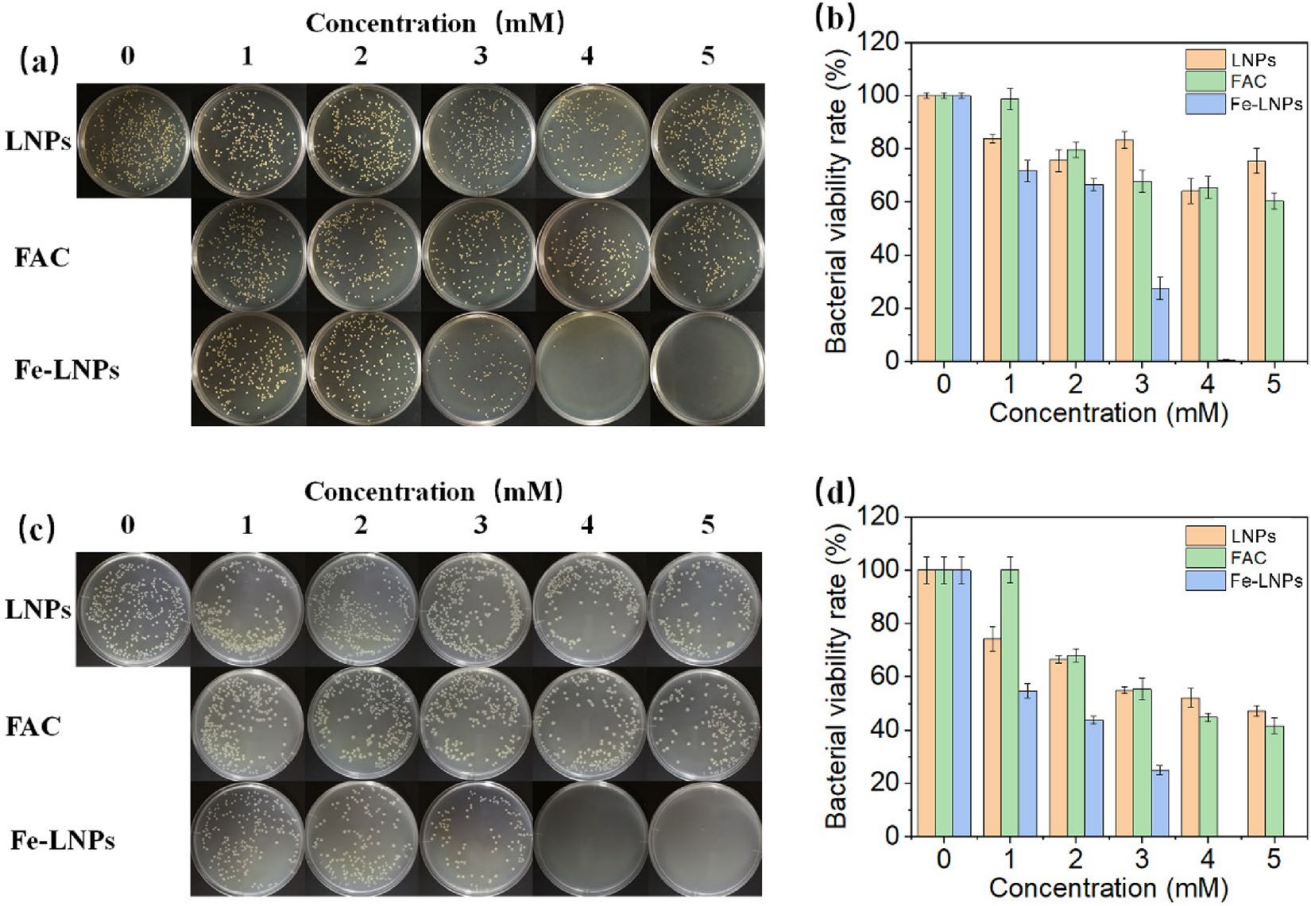
The colony forming pictures (a and c) and corresponding quantitative statistics results (b and d) of *S. aureus* (a and b) and *E. coli* (c and d) after treated with different formulations at different concentrations (FAC).

Afterward, we tested the antibacterial performance of Fe-LNPs on both Gram-positive and negative strains. To exclude the potential interferences, free LNPs and FAC solutions with the same concentrations were also employed as controls. The relative bacterial viability rate was calculated to compare with the untreated group. The pictures of colonies formed at the end of the studies are presented in Figure 2(a,c) while the corresponding quantitative statistics results are displayed in Figure 2(b,d). As shown in Figure 2, although all three formulations show concentration-dependent inhibition on the growth of both strains compared to the untreated group, it is clearly demonstrated that Fe-LNPs exert significantly improved antibacterial effects than FAC and LNPs. Therefore, it is inferred that the combination of both elements is the key for the effective killing performance. FAC is a hydrophilic molecule which cannot readily penetrate to the cytoplasm of bacteria due the selectiveness of lipid bilayer structure of cell membrane. In contrast, previous cellular uptake assays have confirmed that LNPs with similar components to the cell membrane can readily adhere to and being internalized by cells (Yu et al., 2014; S.Q. Chen et al., 2018). Therefore, Fe-LNPs are suggested to increase the intracellular iron concentration by taking advantage of the high cell compatibility nature of LNPs. In detail, the IC90 for both strains under the given condition is around 4 mM (FAC concentration). Therefore, this concentration is selected in the following assays unless otherwise stated. From these results, we can also conclude that Fe-LNPs are promising platform which can give equal inhibition on both Gram-positive and negative stains. This is an exciting merit which can be further extended to be a universally applied antibacterial formulation.

**Figure 3.**
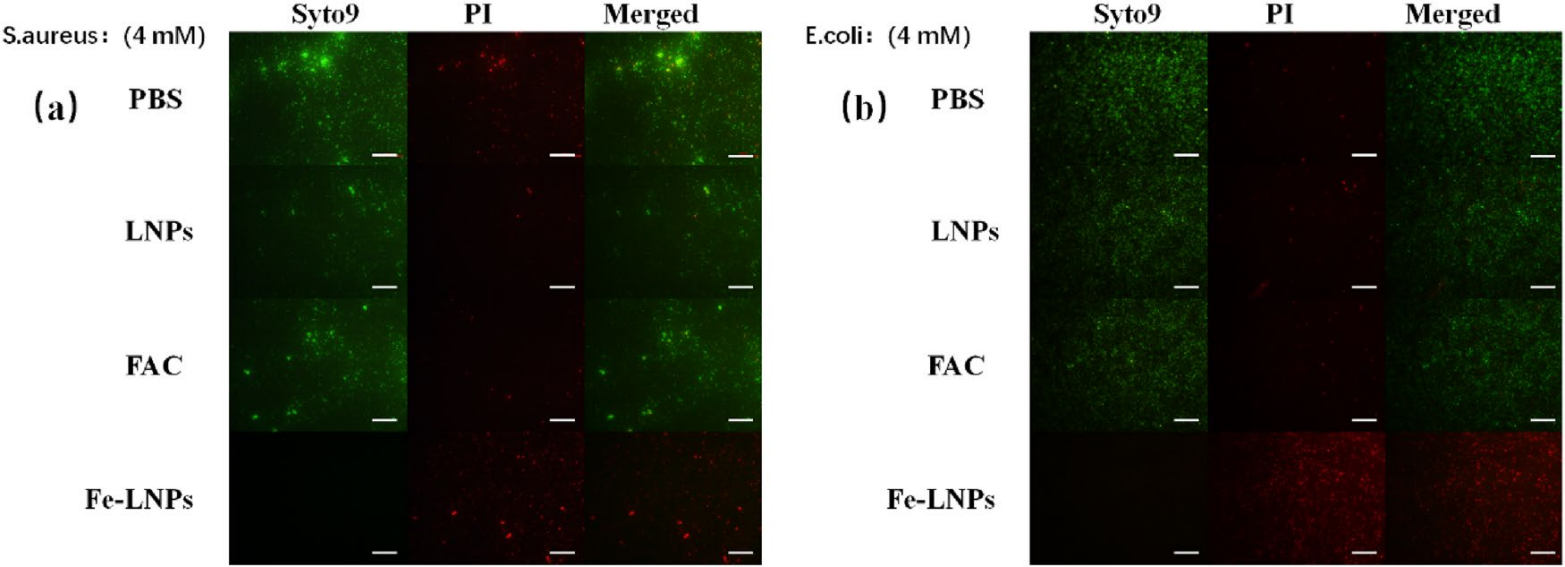
The live/dead staining of *S. aureus* (a) and *E. coli* (d) after treated with different formulations (FAC concentration of 4 mM). Scale bar is 100 μm.

Next, the live/dead staining was employed as to visualize the killing effect of Fe-LNPs. Syto9 is a highly permeable probe that can bind with nucleic acid of both live and dead cells to give green fluorescence signal. In contrast, PI can only penetrate into bacteria with compromised cell membranes and bind with nucleic acid to give red fluorescence signal. It was noted that the insertion of PI into the same nucleic acid can reduce the signal of Syto9, which finally give the results that living bacteria are shown with green fluorescence while dead ones are stained by PI to give red fluorescence under fluorescence microscope. As shown in Figure 3, in contrast to other groups that have observable green dots (living cells) in the view, after treated with 4 mM of Fe-LNPs, most of the bacteria are suffered from severe cell membrane damage and most the cells are stained by PI to give red fluorescence, indicating significant loss of viability. This effect can might be associated with the ROS generated by fenton reaction as lipid peroxidation often gives damage to the integrity of membrane structure. Moreover, these results are in consistent with that obtained in Figure 2, which further prove the powerful inhibition effects of Fe-LNPs on both strains.

**Figure 4.**
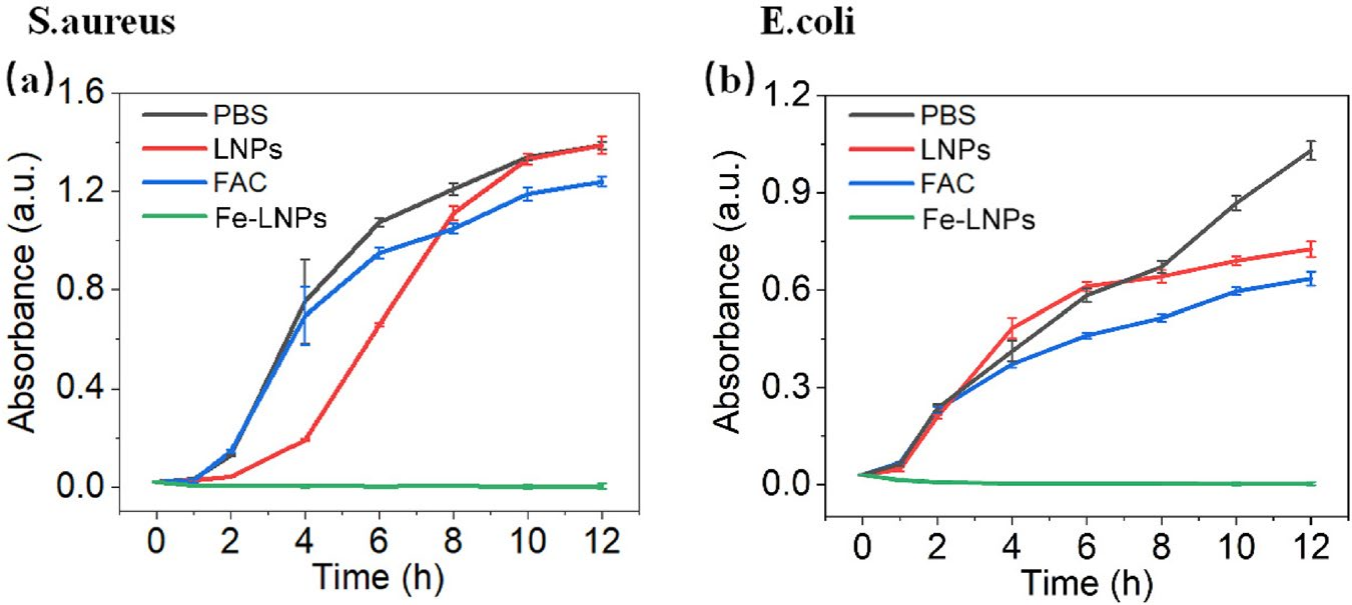
The time-dependent growth curve of *S. aureus* (a) and *E. coli* (b) after treated with different formulations.

As another proof, the growth curves of both strains incubating with different formulations are recorded by monitoring the OD_600_ of bacteria suspension at pre-arranged time points. As displayed in Figure 4, in control groups (PBS, FAC, and LNPs), both strains show persistent increase on OD_600_ during the whole testing period, suggesting weak antibacterial effect of FAC and LNPs. However, upon treating with 4 mM of Fe-LNPs, there is no increase on OD_600_ even at 12 h postincubation, suggesting the full inhibition of bacteria. These results were in line with that obtained in Figure 2 to further confirm the powerful antibacterial effects of Fe-LNPs. Based on the these results, it was suggested that Fe-LNPs might be further developed as long-acting dressings for persistent disfection of wounds.

**Figure 5.**
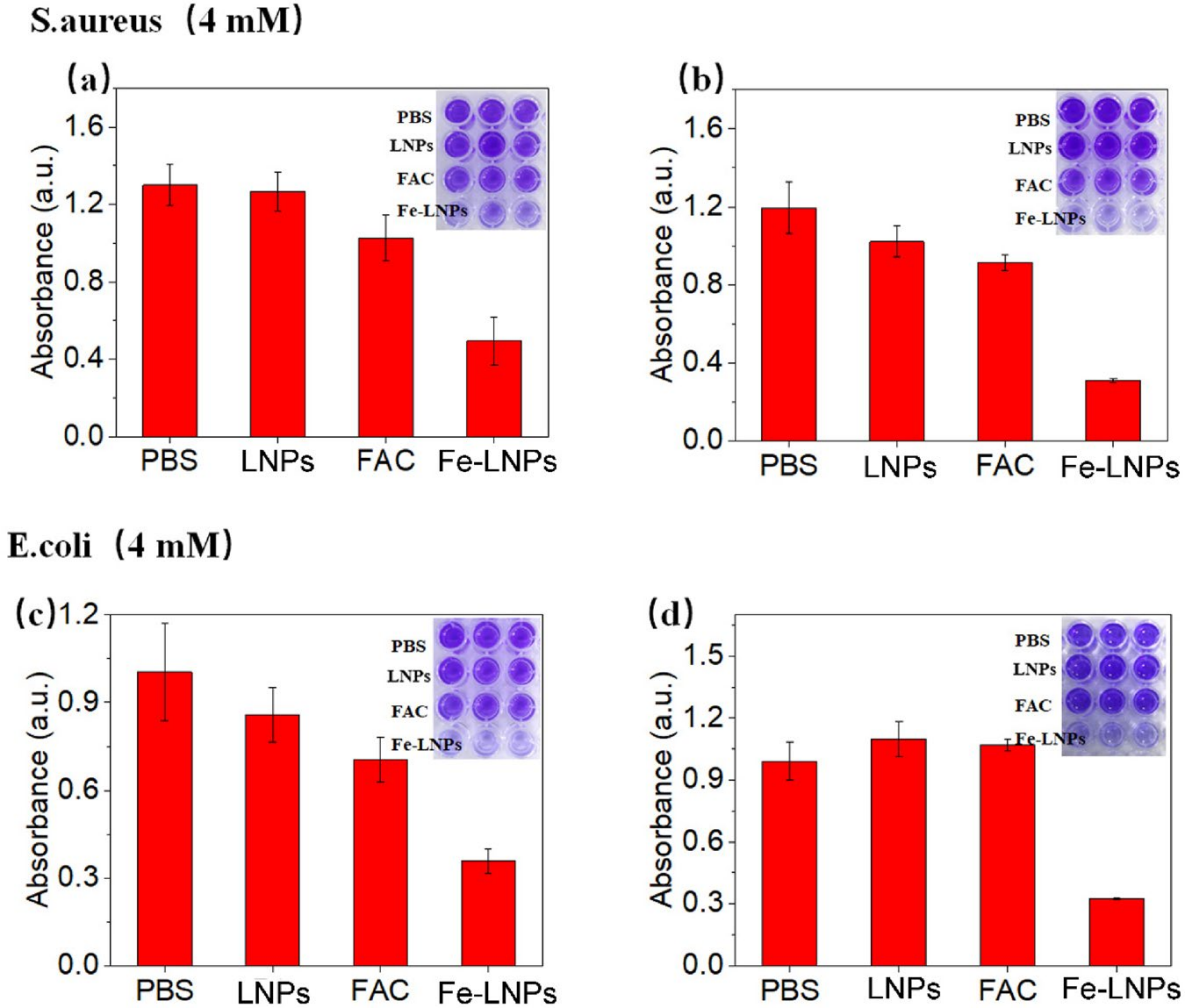
The inhibition (a and c) and destruction (b and d) of *S. aureus* (a and b) and *E. coli* (c and d) after treated with different formulations.

A biofilm is generally recognized as a living bacterial population or community with organized structures that locate at a liquid interface. Biofilms are shown to have higher resistance to antibacterial drugs than individual bacteria (Davies, 2003). Furthermore, biofilms can also help the encapsulated bacteria to evade the host defense systems (Vestby et al., 2020). Therefore, the ability to prevent biofilm formation and destroy the formed ones is critical for formulations intended to be applied as promising antibacterial formulation (Liang et al., 2022). Here in this study, the biofilm prevention and destruction abilities of Fe-LNPs are tested on both strains. Fe-LNPs and other formulations were added to the bacterial suspension and incubated for 48 h. The formation of biofilm upon different treatments was tested using crystal violet staining. As shown in Figure 5(a,c), FAC and LNPs only show minor inhibition on the formation of biofilms while Fe-LNPs show significantly improved biofilm inhibition effects. In the biofilm destruction assay in Figure 5(b,d), the results are similar to that of biofilm formation assay with Fe-LNPs exerting the most powerful eradicating effects on the formed biofilms. These results demonstrate the powerful biofilm prevention and destruction capability of Fe-LNPs on both strains, which also suggest the wide-spectrum antibacterial potential of Fe-LNPs to serve as an universal material in wound management.

**Figure 6.**
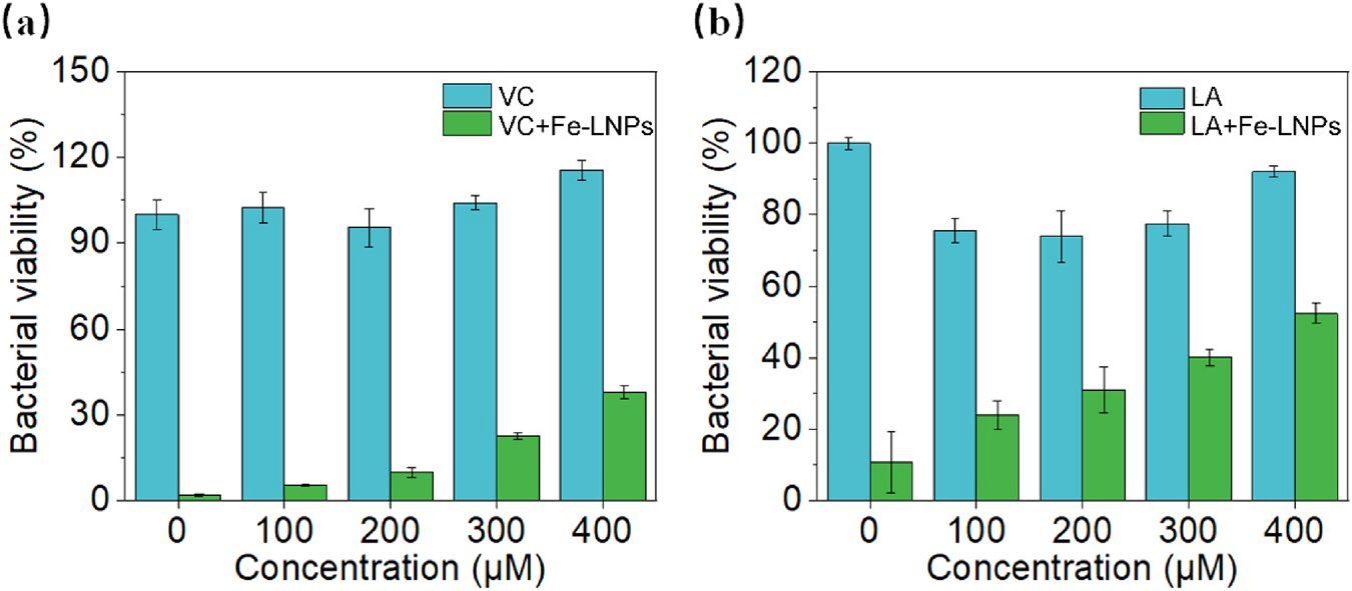
The bacterial viability assay of *S. aureus* after VC (a) and LA (b) treatment.

VC and LA are two commonly used antioxidants that can show beneficial effects in a number of oxidative stress models (Packer et al., 1995). It was reported that LA inhibits the progress of ferroptosis by reducing iron accumulation and lipid peroxidation (Y.-H. Zhang et al., 2018) while the VC suppresses the initiation of ferroptosis by scavenging the free radicals (H.M. Tang & Tang, 2019). To understand whether the antibacterial mechanism of Fe-LNPs is associated with ferroptosis-like cell death, VC and LA were co-incubated with Fe-LNPs to test the changes in bacterial viability. Free VC and LA under the same concentrations were selected as controls. As shown in Figure 6, free VC and LA exert weak inhibition effects on the viability of *S. aureus*, suggesting their low cytotoxicity. Upon co-incubating with Fe-LNPs, we are excited to note that the viability of *S. aureus* recovers with the increasing concentrations of both substances. These results clearly demonstrate that ROS is responsible for the antibacterial effects of Fe-LNPs. Considering the indispensable role of iron in this formulation, we have sufficient reasons to suggest that ferroptosis like cell death is involved in the antibacterial process of Fe-LNPs.

**Figure 7.**
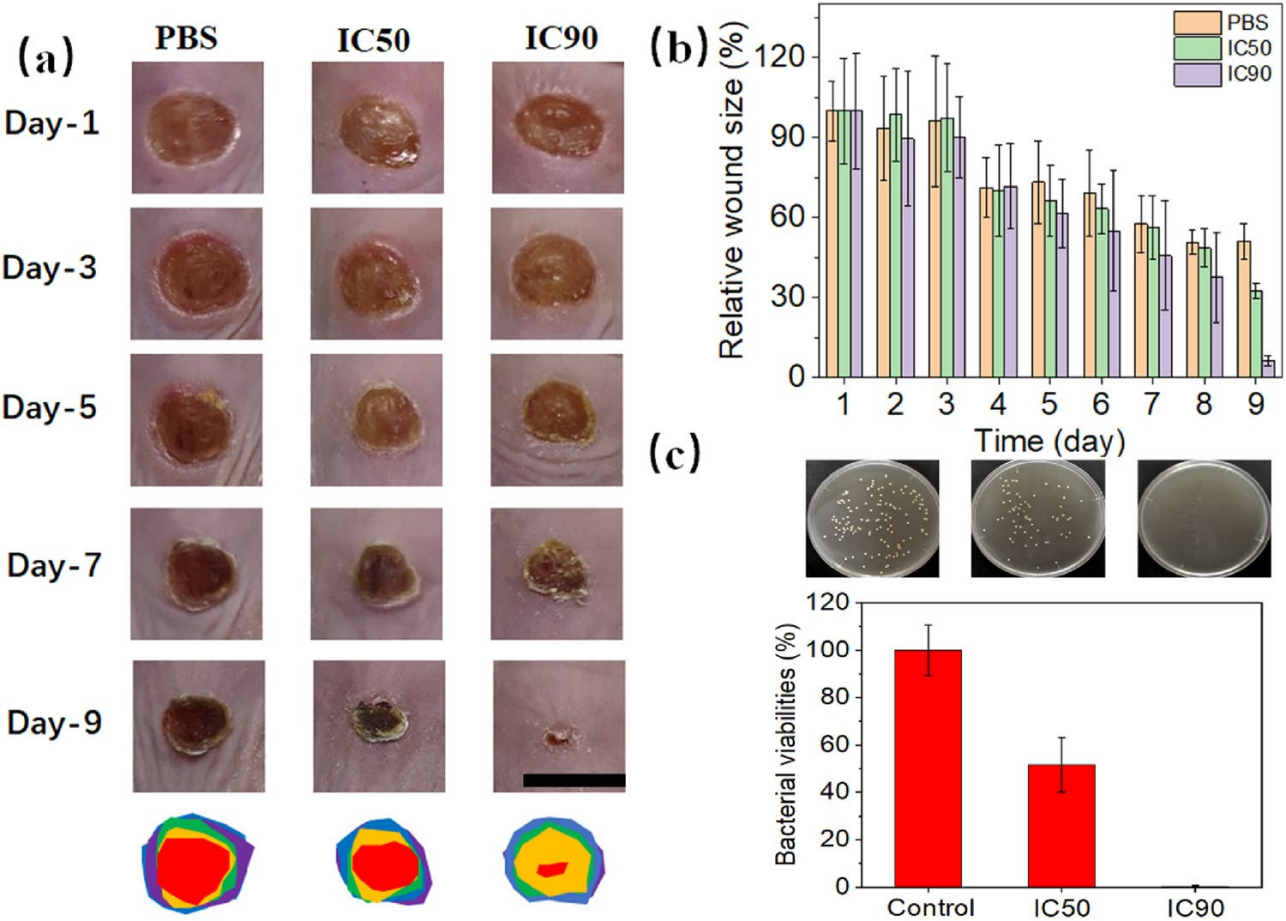
The skin infection management and wound healing promotion effects of Fe-LNPs. (a) The time-dependent picture of wounds in different groups. Scale bar: 1 cm. (b) The quantitative statistics of the relative wound size in different groups. (c) The colony forming and corresponding quantitative statistics results of skin tissue from different groups.

Finally, a *S. aureus* infection model was established and then employed to test the in vivo antibacterial effects of Fe-LNPs. The skin was firstly wounded and inoculated with *S. aureus* suspension. At two days after inoculation, the wounds were treated with PBS, 2 mM of Fe-LNPs (IC_50_), and 4 mM of Fe-LNPs (IC_90_) every day for three times. The start of experiment was defined as Day 1 and the changes in wounds and body weights were recorded every day for 9 days. As shown in Figure 7(a,b), the wounds in PBS group still maintain about 60% of the original size with infections observed at Day 9. On the contrary, treating with Fe-LNPs shows significantly reduced skin infection and improved wound healing in a concentration-dependent manner. In particular, there is only 10% of wound remaining at the end of test in IC90 group. As another proof, the skin tissue of wounds from each group was isolated and homogenated in sterile PBS, followed by colony forming assay to study the remaining bacteria in wounds. As shown in Figure 7(c), the colony forming assay also suggest the large survival of *S. aureus* in wounds treated by PBS while full elimination of *S. aureus* was achieved in wounds treated by 4 mM of Fe-LNPs. These results were not only consistent with the *in vitro* antibacterial results but also collectively confirm the powerful *in vivo* antibacterial effects of Fe-LNPs. It was also found that the wound healing and antibacterial effects of these three groups are positively related, suggesting that wound healing might benefit from the antibacterial effect of formulations.

## 4. Conclusion

In summary, in this study, we proposed the strategy of treating skin infection using ferroptosis and designed a FAC-loaded LNPs (Fe-LNPs) as antibacterial formulation. Our results demonstrate that Fe-LNPs are nanosized particles with average size of 130 nm and surface charge of –12 mV. In the battle against both Gram-positive and negative strains, Fe-LNPs show equal inhibition effect with a IC90 of 4 mM. It was noted that the antibacterial effect of Fe-LNPs can only be realized upon the combination of both FAC and LNPs while single component only shows feeble inhibition effects on both strains. The following live/dead staining, growth curve monitoring assays, and biofilm prevention/inhibition assays also reached consistent results. Importantly, the addition of VC and LA can significantly impair the antibacterial effects of Fe-LNPs, suggesting the ferroptosis like cell death mechanism of Fe-LNPs on bacteria. Finally, Fe-LNPs can also effectively inhibit the proliferation of *S. aureus* in a skin infection model and promote the healing of wounds.
